# Discussion

**Published:** 1908-01

**Authors:** Marvin H. Smith


### DISCUSSION.

---

Dr. Michael Hoke: The paper to which we have just listened has been exceedingly interesting. The leading point brought out shows to us how effective the High Frequency current may be in cases of perforating ulcer of the foot and leads us to see that it can be used to advantage in other similar conditions.

Dr. M.B. Hutchins: I do not remember that I ever saw a case or perforating ulcer of the foot exactly in this location. I am not certain that this is a true perforating ulcer. These ulcers usually occur between the meta-carpo phalangeal joints.

The Doctor's case is evidently traumatic. The treatment in this case was largely stimulative; the electric current stimulated the nerve supply. Another advantage of this treatment is that it is not painful to the patient. I believe that if we could get at the core of this disease, we would see that the whole condition was one that depended upon stimulation. I think that Dr. Campbell is to be congratulated upon his success in his cure of this stubborn case.

Perforating ulcers of the foot are extremely rare. Diseased tissues in these cases seem to have no way of repairing themselves, but in this case Dr. Campbell used what was necessary.

Reported by Marvin H. Smith.

